# Supplementary material for: Cytological Quantification of Nodal Mast Cells in Dogs Affected by Non-Neoplastic Condition and Mast Cell Tumor Using Different Sample Preparation Techniques: An Explorative Study
Source: Animals (Basel). 2023 Aug 15;13(16):2634. doi: 10.3390/ani13162634 (PMC10451199; doi:10.3390/ani13162634)
Supplement: Supplementary file 1 [file animals-13-02634-s001.zip › animals-2525812-supplementary.pdf]

Supplementary Material

Figure S1 – Selection workflow for cases included in the current study.

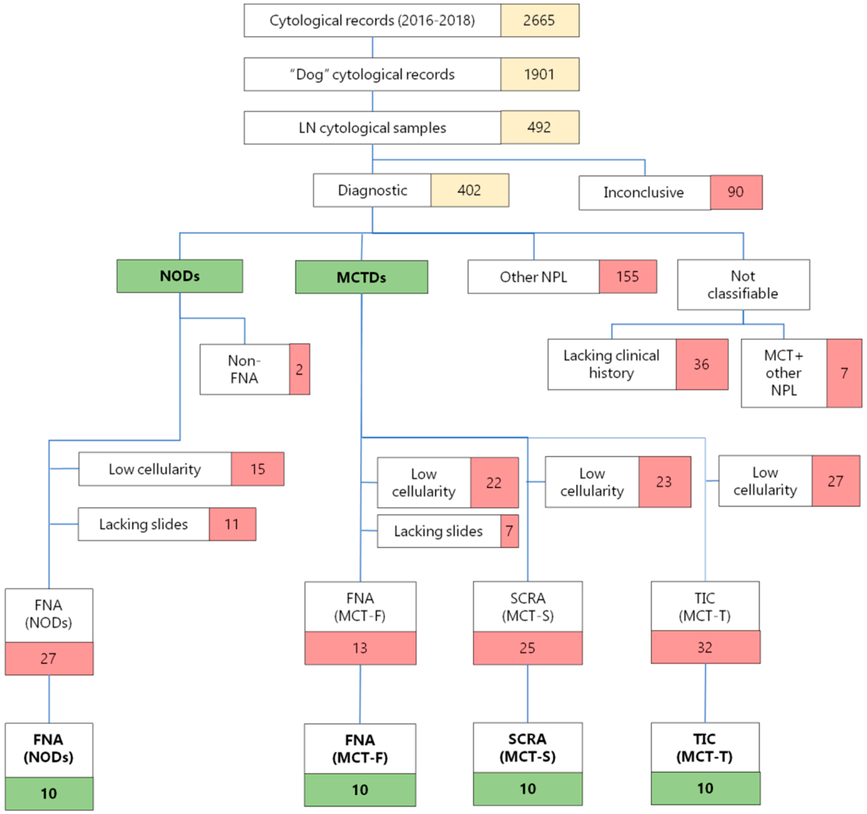

Legend: FNA, fine-needle aspiration; LN, lymph node; MCT, mast cell tumor; MCTDs, mast cell tumor-bearing dogs; NODs, non-oncological dogs; NPL, neoplasm; cellularity; SCRA., scraping smears; TIC, touch imprints.

Figure S2 – Example microphotograph showing the square grid applied to facilitate cell counting (A) and examples of cells excluded from counting (B).

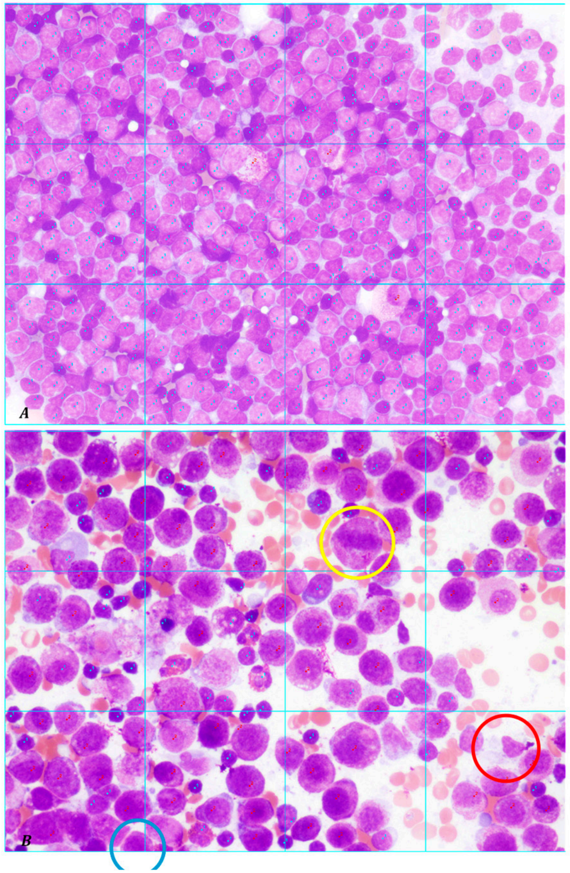

A. Example microphotograph showing the square grid applied to facilitate cell counting. Red dots: mast cells (MCs), blue dots: cells other than MCs.
B. Examples of cells excluded from counting. Encircled in yellow a mitotic cell. Encircled in red, a naked nucleus. Encircled in blue, a cell only partially depicted in the figure.

Table S1 - Signalment data of dogs included in the study and details regarding investigated LNs.

Dog ID	Breed	Sex	Age	Primary process	Case ID	LN location	LN size	RLN or SLN	Category / Subcategory	Histology (agreement)	Retrieved cytological slides	# cytological slides used for micropics	Entire-ly-counted micropics
1	Mongrel	NF	3yy	Rhinitis, dermatitis, and reactive histiocytosis	#8	R mandibular	Megalic (mild)	RLN	NODs	n/a	2	2	5
					#28	L mandibular	Normal	RLN	NODs	n/a	2	2	7
2	Mongrel	CM	9yy	Necrotizing gingivitis	#9	Retromandibular	n/d	RLN	NODs	n/a	2	1	8
3	Dachshund	F	5yy	Bilateral purulent otitis	#35	R prescapular	Megalic (moderate)	RLN	NODs	n/a	2	1	7
					#16	L prescapular	Megalic (moderate)	RLN	NODs	n/a	1	1	6
4	Ibizan Hound	Ma	1yy	Leishmaniasis	#18	L submandibular	Megalic (mild)	RLN	NODs	n/a	2	2	6
5	Dachshund	NF	9yy	Leishmaniasis	#37	Prescapular	3 cm	RLN	NODs	n/a	2	2	8
6	Shar Pei	NF	4yy 8mm	Hepatic amyloidosis	#38	R popliteal	1 cm	RLN	NODs	n/a	1	1	8
7	Mongrel	Ma	9yy	Allergic dermatopathy	#21	n/d	n/d	RLN	NODs	n/a	2	2	13
8	English Setter	NF	12yy	Stomatitis	#2	Cervical (ventral)	n/d	RLN	NODs	n/a	2	2	12
9	Jack Russell Terrier	NF	11yy 11mm	MCT (n/d)	#39	L iliac	Megalic (n/d)	RLN	MCT-M MCT-F	n/a	3	1	14
					#19	R iliac	Megalic (n/d)	RLN	MCT-M MCT-F	n/a	1	1	14

Dog ID	Breed	Sex	Age	Primary process	Case ID	LN location	LN size	RLN or SLN	Category / Subcategory	Histology (agreement)	Retrieved cytological slides	# cytological slides used for micropics	Entire-ly-counted micropics
10	Coton de Tulear	NF	13yy 11mm	MCT (L thorax)	#20	L axillary	n/d	RLN	MCT-M MCT-F	n/a	2	2	19
11	French Bulldog	Ma	7yy	MCT (L forelimb)	#40	L cervical (superficial)	n/d	RLN	MCT-M MCT-F	HN3 (Y)	3	1	13
12	Beagle	Ma	11yy	MCT (n/d)	#17	R prescapular	Megalic (severe)	RLN	MCT-M MCT-F	n/a	4	2	14
13	Tosa Inu	Ma	4yy	MCT (prepuce)	#6	Popliteal	Megalic (moderate)	RLN	MCT-NM MCT-F	n/a	2	2	8
14	Labrador Retriever	Ma	5yy	MCT (R hindlimb)	#34	R prescapular	Megalic (severe)	RLN	MCT-NM MCT-F	n/a	2	2	16
15	Mongrel	NF	7yy 11mm	MCT (L flank)	#1	L inguinal	Megalic (mild)	RLN	MCT-NM MCT-F	HN2 (N)	2	2	10
16	Maltese	CM	5yy	MCT (R thorax)	#27	L popliteal	Megalic (mild)	RLN	MCT-NM MCT-F	n/a	2	2	10
17	Boxer	F	12yy	MCT (breast; L forelimb – III digit)	#36	L prescapular	Megalic (n/d)	RLN	MCT-PM MCT-F	HN2 (P)	3	1	14
18	Mongrel	NF	6yy	MCT (breast)	#14	R inguinal (lateral)	Normal	RLN	MCT-M MCT-S	HN3 (Y)	2	1	11
					#33	R inguinal (medial)	Normal	RLN	MCT-M MCT-S	HN3 (Y)	2	1	6
19	Labrador Retriever	Ma	7yy	MCT (prepuce)	#15	R inguinal	3x0.8x0.5 cm	SLN	MCT-PM MCT-S	HN0 (N)	1	1	9
20	Mongrel	CM	12yy	MCT (head)	#23	L prescapular #2	1.2x0.7x0.4 cm	SLN	MCT-NM MCT-S	HN0 (Y)	2	2	7
					#3	L prescapular #2	1.2x0.7x0.4	SLN	MCT-NM	HN0 (Y)	1	1	8

Dog ID	Breed	Sex	Age	Primary process	Case ID	LN location	LN size	RLN or SLN	Category / Subcategory	Histology (agreement)	Retrieved cytological slides	# cytological slides used for micropics	Entire-ly-counted micropics
							cm		MCT-T				
					#24	L prescapular #1	2.45x1.1x0.95 cm	SLN	MCT-NM MCT-T	HN2 (N)	1	1	6
					#22	L prescapular #3	2.4x1.3x0.6 cm	SLN	MCT-NM MCT-T	HN0 (Y)	1	1	10
				MCT (breast)	#4	R inguinal	1.35x0.95x0.45 cm	SLN	MCT-M MCT-S	HN2 (Y)	1	1	7
					#5	R inguinal	1.35x0.95x0.45 cm	SLN	MCT-PM MCT-T	HN2 (P)	1	1	9
				MCT (R thight)	#25	R inguinal #2	2.5x1x0.7 cm	SLN	MCT-PM MCT-S	HN2 (P)	1	1	8
					#26	R inguinal #2	2.5x1x0.7 cm	SLN	MCT-M MCT-T	HN2 (Y)	1	1	9
21	English Setter	M	6yy	MCT (R hindlimb)	#10	R inguinal #1	1.8x1.5 cm	SLN	MCT-M MCT-S	HN3 (Y)	1	1	12
					#30	R inguinal #2	0.9x1 cm	SLN	MCT-M MCT-S	HN3 (Y)	1	1	19
					#31	R popliteal	3.1x1.4 cm	SLN	MCT-PM MCT-T	HN2 (P)	1	1	7
22	Mongrel	NF	7yy	MCT (n/d)	#32	Prescapular	Normal	SLN	MCT-M MCT-S	HN3 (Y)	2	1	16
					#11	Prescapular	Normal	SLN	MCT-M MCT-T	HN3 (Y)	2	1	8
23	Boxer	NF	6yy	MCT (L forelimb)	#12	L prescapular	n/d	RLN	MCT-NM MCT-S	HN3 (N)	4	4	5
					#13	L prescapular	n/d	RLN	MCT-M MCT-T	HN3 (Y)	5	2	6

Dog ID	Breed	Sex	Age	Primary process	Case ID	LN location	LN size	RLN or SLN	Category / Subcategory	Histology (agreement)	Retrieved cytological slides	# cytological slides used for micropics	Entire-ly-counted micropics
24	Mongrel	CM	4yy	MCT (L thorax)	#7	Axillary (acces-sory)	0.5x0.5x0.3 cm	SLN	MCT-NM MCT-T	HN0 (Y)	2	2	8
25	Golden Retriever	NF	4yy	MCT (R hock)	#29	R popliteal	n/d	RLN	MCT-M MCT-T	HN3 (Y)	5	4	12

Legend: Case ID, randomized blinded ID assigned to each cytological specimen considered as an independent case; CM, castrated male; F, female; HNO, "non-metastatic" according to Weishaar et al.;[17] HN2, "early metastasis" according to Weishaar et al.; HN3, "overt metastasis" according to Weishaar et al.; L, left; LN, limph node; Ma, male; MCT, mast cell tumor; MCT-F, nodal sample obtained from MCT-bearing dog via fine-needle aspiration; MCT-M, cytologically metastatic nodal sample obtained from MCT-bearing dog; MCT-NM, cytologically non metastatic nodal sample obtained from MCT-bearing dog; MCT-PM, cytologically possibly metastatic nodal sample obtained from MCT-bearing dog; MCT-S, nodal sample obtained from MCT-bearing dog via scraping smearing; MCT-T, nodal sample obtained from MCT-bearing dog via touch imprinting; Megalic (mild), <2cm; Megalic (moderate), 2-5cm; Megalic (severe), i.e. >5cm; micropic, microphotographs; mm, months; NF, neutered female; NODs, nodal sample obtained from non-oncological dog; n/a, not applied; n/d, not determined; R, right; RLN, regional lymph node; SNL, sentinel lymph node; yy, years; (N), lack of agreement between cytological and histological diagnosis; (P), partial agreement between cytological and histological diagnosis; (Y), agreement between cytological and histological diagnosis.

Cytological and histological diagnosis were considered in agreement when a MCT-NM corresponded to HN0, a MCT-PM corresponded to HN1, and a MCT-M corresponded to HN2 or HN3. Cytological and histological diagnosis were considered in partial agreement when a MCT-PM corresponded to HN2 or HN3.

Table S2 – Median and mean absolute and percentage number of nodal mast cells in non-oncological dogs (NODs) and mast cell tumor-bearing dogs (MCTDs).

Group	# cases	Counting method	Median	Min-max range	Mean	Std dev	Std err	Err coeff	Median%	Min-max range%	Mean%	Std dev%
**NODs**	10	4 HPFs	0	0-1	0.1	0.32	0.1	n/a	//	//	//	//
		8 HPFs	0	0-2	0.2	0.63	0.2	n/a	//	//	//	//
		20 HPFs	0.5	0-11	1.7	3.37	1.7	n/a	//	//	//	//
		500 cells	0	0	0	0	0	n/a	0	0	0	0
		1000 cells	0	0-0.86	0.09	0.27	0.09	n/a	0	0-0.09	0.01	0.03
		2000 cells	0	0-2	0.2	0.63	0.2	n/a	0	0-0.1	0.01	0.04
**MCTDs**	30	4 HPFs	8	0-511	85.6	130.5	23.82	0.28	//	//	//	//
		8 HPFs	13.5	0-1073	173.1	268.6	49.03	0.28	//	//	//	//
		20 HPFs	28	1-2362	428.3	657.2	119.98	0.28	//	//	//	//
		500 cells	3.90	0-427.9	79.22	130.2	23.78	0.3	0.78	0-85.58	15.84	26.05
		1000 cells	6.06	0-877.1	153.8	257.5	47.01	0.31	0.61	0-87.71	15.38	25.75

**Group**	**# cases**	**Counting method**	**Median**	**Min-max range**	**Mean**	**Std dev**	**Std err**	**Err coeff**	**Median%**	**Min-max range%**	**Mean%**	**Std dev%**
		2000 cells	12.8	0-1743	307.9	513.6	93.77	0.3	0.64	0-87.14	15.39	25.68

Legend: Err coeff, error coefficient (calculataed as the standard error of Mean divided by Mean); HPFs, high power fields; Median%, median of the number of mast cells expressed as the percentage over 500, 1000, and 2000 cells; Min-max range, minimum-mximum range referred to the Median; Min-max range%, minimum-maximum range referred to the Median%; Mean%, mean of the number of mast cells expressed as the percentage over 500, 1000, and 2000 cells; n/a, not applied; Std dev, standard deviation; Std dev%, standard deviation referred to the Mean%; Std err, standard error of Mean.

animals

MDPI

Table S3 – Median and mean absolute and percentage number of mast cells in nodal specimens obtained from mast cell tumor-bearing dogs (MCTDs) and classified as "non metastatic" (MCT-NM), "possibly metastatic" (MCT-PM), and "metastatic" (MCT-M).

Group	# cases	Counting method	Median	Min-max range	Mean	Std dev	Median%	Min-max range%	Mean %	Std dev %
MCT-NM	10	4 HPFs	0	0-3	0.6	0.97	//	//	//	//
		8 HPFs	0.50	0-4	1.10	1.45	//	//	//	//
		20 HPFs	2	1-6	2.40	1.58	//	//	//	//
		500 cells	0	0-0.9	0.25	0.40	0	0-0.18	0.05	0.08
		1000 cells	0	0-2.6	0.68	1.02	0	0-0.26	0.07	0.10
		2000 cells	0.50	0-2.91	1.04	1.22	0.03	0-0.15	0.05	0.06
MCT-PM	5	4 HPFs	5	1-9	5.40	2.97	//	//	//	//
		8 HPFs	9	3-16	8.80	5.12	//	//	//	//
		20 HPFs	28	6-28	19.40	11.78	//	//	//	//
		500 cells	4.39	0.97-6.38	4.05	2.26	0.88	0.19-1.28	0.81	0.45
		1000 cells	5.24	1.99-7.54	5.12	2.24	0.52	0.20-0.75	0.51	0.22
		2000 cells	8.70	4.84-16.82	9.39	5.02	0.43	0.24-0.84	0.47	0.25
MCT-M	15	4 HPFs	188	5-511	169	142.7	//	//	//	//
		8 HPFs	319	7-1073	342.6	296.4	//	//	//	//
		20 HPFs	782	21-2362	848.6	718.3	//	//	//	//
		500 cells	120	0.88-42	156.9	149	24	0.18-85.	31.39	29.79

**Group**	**# cases**	**Counting method**	**Median**	**Min-max range**	**Mean**	**Std dev**	**Median%**	**Min-max range%**	**Mean %**	**Std dev %**
				7.9				58		
		1000 cells	230.8	4.34-877.1	305.4	296.7	23.08	0.43-87.71	30.54	29.67
		2000 cells	525.3	6.98-1743	611.9	590.2	26.26	0.35-87.14	30.60	29.51

Legend: HPFs, high power fields; Median%, median of the number of mast cells expressed as the percentage over 500, 1000, and 2000 cells; Min-max range, minimum-mximum range referred to the Median; Min-max range%, minimum-maximum range referred to the Median%; Mean%, mean of the number of mast cells expressed as the percentage over 500, 1000, and 2000 cells; Std dev, standard deviation; Std dev%, standard deviation referred to the Mean%.

Table S4 – Median and mean absolute and percentage number of mast cells in nodal specimens obtained from mast cell tumor-bearing dogs (MCTDs) and sampled via fine-needle aspiration (MCT-F), scraping smearing (MCT-S), and touch imprinting (MCT-T).

Group	# cases	Counting method	Median	Min-max range	Mean	Std dev	Medi-an%	Min-max range%	Mean %	Std dev%
MCT-F	10	4 HPFs	94.5	0-511	156.3	182.6	//	//	//	//
		8 HPFs	181.5	0-1073	328.4	385.5	//	//	//	//
		20 HPFs	620	1-2361	833.6	931.7	//	//	//	//
		500 cells	124.4	0-427.9	165.2	181.8	24.89	0-85.58	33.04	36.37
		1000 cells	251.3	0-877.1	332.5	366.1	25.13	0-87.71	33.25	36.61
		2000 cells	476.9	0-1743	652.1	726.1	23.85	87.14	32.61	36.30
MCT-S	5	4 HPFs	26	0-215	73.5	88.93	//	//	//	//
		8 HPFs	55.50	0-426	145.4	168.9	//	//	//	//
		20 HPFs	136.5	2-958	365.8	413.6	//	//	//	//
		500 cells	19.10	0-250	57.83	82.82	3.82	0-50	11.57	16.56
		1000 cells	36.55	0-389.5	102.6	138.5	3.66	0-38.95	10.26	13.86
		2000 cells	66.11	0-886.6	226.2	313.1	3.31	0-44.33	11.31	15.65
MCT-T	15	4 HPFs	7	0-208	27	63.97	//	//	//	//
		8 HPFs	10.50	0-319	45.60	97.88	//	//	//	//
		20 HPFs	17.50	1-610	85.60	187.2	//	//	//	//
		500 cells	2.25	0-120	14.66	37.22	0.45	0-24	2.93	7.44
		1000 cells	4.32	0-201.2	26.22	62.01	0.44	0-20.12	2.62	6.20
		2000 cells	9.42	0-309.7	45.29	95.03	0.47	0-15.49	2.27	4.75

Legend: HPFs, high power fields; Median%, median of the number of mast cells expressed as the percentage over 500, 1000, and 2000 cells; Min-max range, minimum-mximum range referred to the Median; Min-max range%, mini-

mum-maximum range referred to the Median%; Mean%, mean of the number of mast cells expressed as the percentage over 500, 1000, and 2000 cells; Std dev, standard deviation; Std dev%, standard deviation referred to the Mean%.

Table S5 – Median and mean cellularity per field of each category and subcategory (i.e. "group") investigated in the current study.

Group	# cases	Median	Min-max range	Mean	Std dev
**NODs**	10	286.7	144.6-407.2	282.1	78.52
**MCTDs**	30	222.2	74.8-448.6	222.5	87.67
**MCT-NM**	10	251.8	144.2-448.6	260.9	87.48
**MCT-PM**	5	238.6	125.2-301.4	231.4	65.59
**MCT-M**	15	162.2	74.8-357.8	193.9	88.40
**MCT-F**	10	147.2	74.80-230.4	153.5	53.20
**MCT-S**	10	245.8	108.8-448.6	249.7	101.5
**MCT-T**	10	281.7	155.6-342.6	264.2	59.86

Legend: FNA, fine-needle aspiration; M, metastatic; MCTDs, mast cell tumor-bearing dogs; MCT-F, nodal sample obtained from MCT-bearing dog via fine-needle aspiration; MCT-M, cytologically metastatic nodal sample obtained from MCT-bearing dog; MCT-NM, cytologically non metastatic nodal sample obtained from MCT-bearing dog; MCT-PM, cytologically possibly metastatic nodal sample obtained from MCT-bearing dog; MCT-S, nodal sample obtained from MCT-bearing dog via scraping smearing; MCT-T, nodal sample obtained from MCT-bearing dog via touch imprinting; Min-max range, minimum-maximum range referred to the Median; NM, non metastatic; NODs, non-oncological dogs; PM, possibly metastatic; SCRA, scraping smearing; Std dev, standard deviation; TIC, touch imprinting.
